# Exploring the Role of ADCs in Brain Metastases and Primary Brain Tumors: Insight and Future Directions

**DOI:** 10.3390/cancers17091591

**Published:** 2025-05-07

**Authors:** Francesco Bruzzone, Chiara Barigazzi, Antonio Di Muzio, Isabel Tallarico, Angelo Dipasquale, Agnese Losurdo, Pasquale Persico, Pierina Navarria, Federico Pessina, Armando Santoro, Matteo Simonelli

**Affiliations:** 1 Department of Medical Oncology and Hematology Unit, IRCCS Humanitas Research Hospital, Rozzano, 20089 Milan, Italy; francesco.bruzzone@humanitas.it (F.B.); chiara.barigazzi@humanitas.it (C.B.); antonio.dimuzio@humanitas.it (A.D.M.); isabel.tallarico@humanitas.it (I.T.); angelo.dipasquale@humanitas.it (A.D.); agnese.losurdo@humanitas.it (A.L.); pasquale.persico@hunimed.eu (P.P.); armando.santoro@humanitas.it (A.S.); 2 Department of Biomedical Sciences, Humanitas University, Pieve Emanuele, 20072 Milan, Italy; federico.pessina@hunimed.eu; 3 Department of Radiotherapy and Radiosurgery, IRCCS Humanitas Research Hospital, Rozzano, 20089 Milan, Italy; pierina.navarria@humanitas.it; 4 Department of Neurosurgery, IRCCS Humanitas Research Hospital, Rozzano, 20089 Milan, Italy

**Keywords:** antibody–drug conjugates (ADCs), lung cancer, radiotherapy, breast cancer, neuro-oncology, targeted therapies

## Abstract

Primary and secondary brain tumors have always been a challenge due to their high morbidity and poor prognosis. The incidence of brain metastasis is also increasing with the advent of effective new treatments. Traditional systemic treatments have historically had limited success, partly due to poor central nervous system (CNS) penetration. However, the advent in recent decades of new therapies that have shown high encephalic response rates are challenging this paradigm. ADCs represent a new class of compounds revolutionizing cancer treatment with high systemic response rates and lower toxicities. The continuing evolution of ADCs has shown that certain structural features such as payload, linker, and drug-to-antibody ratio (DAR) are essential in determining their efficacy at the encephalic level, and some ADCs have started to exhibit promising efficacy in treating primary and secondary brain tumors. Unfortunately, most patients with untreated encephalic metastases are excluded from clinical trials, with data primarily from retrospective studies or post hoc analyses. This review describes the early signs of ADC efficacy in brain tumors, the role of complementary treatments like radiation therapy, and critical points to improve ADC efficacy in brain malignancies.

## Simple Summary

Primary and secondary brain tumors are historically treated with loco-regional approaches such as surgery or radiation therapy. Recently, new pharmacological agents like tyrosine kinase inhibitors (TKIs) have challenged this paradigm due to high intracranial response rates in patient subgroups. Drug-conjugated antibodies (ADCs), such as trastuzumab deruxtecan, represent a new class of compounds increasingly used in systemic cancer treatment. This review aims to analyze the efficacy of ADCs in treating primary and secondary brain tumors, while exploring strategies to enhance their effectiveness. These findings highlight strategies to enhance ADCs’ intracranial efficacy and the need to refine the design of clinical trials including patients with intracranial untreated metastases.

## 1. Introduction

Primary and secondary brain tumors still remain a significant challenge in modern oncology due to their high morbidity and poor prognosis [1,2].

The overall annual cumulative incidence of primary malignant CNS tumors in the US is approximately 6.94 per 100,000 person-years [3]. Among them, glioblastoma (GBM) represents the most common and deadly form in adults, with a 5-year overall survival (OS) rate of 7.2% despite the aggressive multidisciplinary approach used to treat it, including surgery, radiotherapy (RT) and chemotherapy [4].

Brain metastases (BMs) are ten times more common than primary brain tumors (pBTs) [5], with approximately 20% of patients with advanced solid tumors developing BMs during their lifetime, particularly those with lung and breast cancers and melanoma [6].

Despite the use of surgery, RT, and chemotherapy, and the recent advent of novel effective agents such as immunotherapy and targeted therapy, the prognosis of patients with BMs remains generally poor [7]. Moreover, CNS malignancies are often highly symptomatic, with a devastating impact on patients’ quality of life, further compromised by the potential side effects of radiation and surgical treatments [8,9].

Currently, BMs are primarily managed with local treatments such as RT and, in selected cases, surgery. Radiotherapy approaches have evolved over time, transitioning from traditional whole-brain RT to more accurate stereotactic radiosurgery, leading to better disease control while minimizing neurocognitive side effects [10].

Historically, the effectiveness of systemic treatment for CNS tumors has been limited by the presence of the blood–brain barrier (BBB) [11], which is responsible for maintaining the homeostasis of CNS but often impedes adequate drug penetration and the bioavailability of the most used anticancer agents.

The BBB consists of a layer of endothelial cells that surround the blood vessels, supported by cells such as astrocytes and pericytes. Endothelial cells encircling vessels in the CNS differ from peripheral endothelial cells, as they lack fenestrations and are bound by both tight junctions and adherens junctions that prevent molecules from entering the encephalic circulation. The BBB is equipped with a series of transport systems that facilitate the entry of nutrients and other substances. Small (<400–500 Da) and highly lipophilic molecules can passively cross the BBB thanks to their physicochemical properties, while molecules >550 Da such as monoclonal antibodies (mAbs) need energy-dependent transport systems such as the receptor-mediated transcytosis (RMT). Active efflux mechanisms at the BBB level complicate the CNS penetrance of various compounds even further. Among these, the most well known is P glycoprotein (P-gp), an ATP-dependent efflux transporter highly expressed at the BBB, the function of which is to expel harmful substances from the CNS circulation, including several chemotherapeutic agents, which further limits their accumulation in the CNS. [12]

However, in the presence of growing tumors, the BBB is often altered and disrupted; on the other hand, the blood–tumor barrier (BTB) [13], which is highly heterogeneous but generally more permeable than the BBB, allows larger molecules to traffic into CNS parenchyma [11,14,15].

High-grade gliomas often exhibit a dysfunctional BTB in the central regions, leading to contrast enhancement on T1-weighted MRI sequences, while the peripheral regions, where the tumor exhibits infiltrative growth, maintain an intact BBB. Compared to pBTs, metastatic lesions typically show a more consistent disruption of the BBB and, generally, no infiltrative growth, which facilitates diagnostic imaging and, when feasible, surgical resection. The suboptimal and highly variable drug accumulation in brain tumors mainly depends on the BTB’s heterogeneous permeability for small and large molecules as well as on the heterogeneous perfusion.

The new wave of anticancer agents developed over the last two decades, such as TKIs targeting tumor driver genetic alterations and immune checkpoint inhibitors (ICI), which have revolutionized the treatment paradigm of most cancers recently, has exhibited an unprecedented level of activity in the CNS. Currently the effectiveness of these compounds challenges, at least in some clinical scenarios, the old paradigm of BM treatment management based on local interventions.

ADCs represent an innovative class of antineoplastic drugs that allow the selective delivery of cytotoxics into tumor cells [16], and they are quickly emerging as part of a major breakthrough in cancer therapy in recent years.

As of now, there are only seven ADCs that have received FDA approval for the treatment of solid tumors (Table 1).

**Figure 1:**
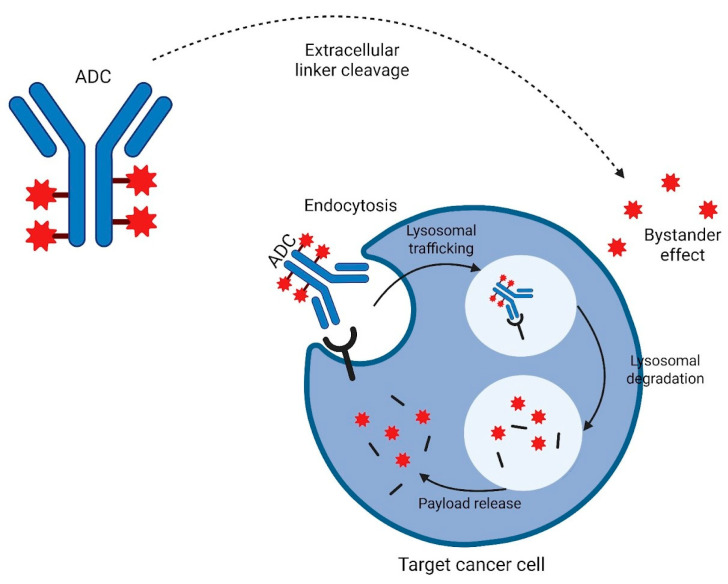
Mechanism of action of ADCs. Abbreviations: ADC, antibody–drug conjugate.

An ADC consists of three main components: the antibody, the linker, and the payload. Linkers can be distinguished into cleavable and non-cleavable linkers. In the case of cleavable linkers, the antibody binds to a target molecule on the tumor cell’s surface, at which point the linker is degraded by conditions in the tumor microenvironment, leading to internalization of the drug. Non-cleavable linkers, in contrast, require endocytosis and degradation of the entire ADC structure to release the payload inside the cell. The resulting cell death enables the payload to affect nearby tumor cells, even those not expressing the target, through a mechanism called the bystander effect (Figure 1) [17].

Non-cleavable linkers are more stable in the bloodstream, but due to their lower cell permeability, they limit the bystander effect, which is crucial when dealing with the heterogeneous expression of the targeted antigen within the tumor [18].

The structural characteristics of an ADC can drastically change the activity of the compound, leading to different levels of efficacy between antibodies, even in the presence of the same target [19].

A crucial factor that determines the effectiveness of ADCs is the DAR, which is the number of chemotherapy molecules carried per single antibody. The DAR can affect ADCs’ stability, solubility, and pharmacodynamics, resulting in different efficacies. A higher DAR might result in better cytotoxicity and an increased bystander effect but lead to more rapid clearance.

Molecules as large as ADCs traditionally cannot cross an intact blood–brain barrier.

However, this paradigm is being challenged by the new generation of ADCs, which have demonstrated high rates of intracranial activity, especially in lung and breast cancer, thus suggesting good brain penetrance. However, the same level of intracranial activity was not observed in the context of primary brain tumors: depatuxizumab mafodotin (Depatux-M), despite its promises, has failed to demonstrate efficacy in patients with EGFR-amplified GBM in both the adjuvant and recurrent treatment settings. In 2021, preclinical studies by Marin et al. showed how the inefficacy of Depatux-M is likely due to insufficient uptake at the encephalic level [20]. This difference in efficacy may be explained by both the differing vascularization and destruction of the BBB between pBTs and BMs.

In addition to intrinsic factors of the BBB, structural features of ADCs, including homogeneity, hydrophobicity, DAR and payload type, seem to be key determinants in the encephalic distribution.

In this comprehensive review, we will thoroughly explore and analyze current evidence on the intracranial activity of ADCs in pBTs and BMs from different primary cancers, including only those tumor types for which there is in a sufficient amount of data in the literature. Despite the current lack of evidence supporting the efficacy of ADCs in the treatment of pBTs, we included a section about the different trials that have been conducted in this setting, from the first attempts with immunotoxins, to the latest INTELLANCE-1 trial, the only phase III study published in this context as of today.

We will further discuss efforts to increase ADC delivery into the brain, the role of radiotherapy as well as biomarkers that could guide us in selecting the optimal treatment for our patients.

The study selection process highlighted that, beyond the inherent biological complexities, substantial methodological challenges persist in designing high-quality systemic therapy trials for patients with brain metastases (BMs) and primary brain tumors (pBTs). These challenges include modernizing eligibility criteria, the use of suboptimal CNS-specific endpoints, inadequate consideration of efficacy denominators and prior radiation exposure, inconsistent application of disease measurability criteria (e.g., RANO, RECIST), and persistent confusion regarding the capture and differentiation of CNS-specific versus extra-CNS efficacy outcomes.

To our knowledge, this is the first narrative review to assess the currently available data on the efficacy of ADCs in both BMs and pBTs and to explore possible strategies to enhance therapeutic effectiveness, including pharmacologic optimization and combination with radiotherapy, with the aim of guiding clinicians toward the most appropriate treatment choices for their patients

Approximately 30 articles presenting clinical trial results published globally between 2007 and 2024 were identified through searches conducted in PubMed, ClinicalTrials.gov, Scopus, and the Cochrane Library. The search utilized keywords including glioblastoma, central nervous system, brain metastases, antibody–drug conjugate, linker, blood-brain-barrier, biomarkers, liquid biopsy and their combinations. Titles and abstracts were screened for relevance to this study. Additionally, the reference lists of the selected reviews and articles were examined to identify further relevant sources.

## 2. Primary Brain Tumors

Given the dismal outcomes and the urgent need for more effective therapeutic options, ADCs have been tested for their potential in treating gliomas.

Immunotoxins could be considered the first form of ADCs designed for GBM treatment. These compounds are constituted of toxins derived from bacteria or plants, conjugated to an antibody targeting a tumor antigen. Pseudomonas aeruginosa exotoxin A (PE) and Diphtheria toxin (DT) are two of the most used toxins in this setting due to their ability to inhibit protein synthesis by modifying elongation factor-2 or by direct ribosomal inhibition [21,22]. These agents’ efficacy might be provided by their specificity, potency, immunity to resistance mechanisms, and independence on cell cycle kinetics [23].

The first immunotoxins developed for GBM targeted the epidermal growth factor receptor (EGFR) pathway [24].

The epidermal growth factor receptor variant III (EGFRvIII) is the most common EGFR mutation occurring in approximately 25–64% of GBM cases; it derives from the in-frame deletion of exons 2–7 of the EGFR gene and codes for a constitutively active, oncogenic, ligand-independent receptor. EGFR amplification is also common in GBM, being present in ~50% of cases [25] (35% in the Asian population) [26]. EGFR amplifications co-exist with EGFRvIII in ~50% of cases (~25% of GBM overall) [27].

TP-38 is a recombinant chimeric protein containing a genetically engineered form of the PE whose native binding domain has been replaced with transforming growth factor-α (TGF-α), making it able to specifically target EGFR [23]. A phase I study of TP-38 administered to patients with progressive or recurrent primary or metastatic brain tumors (including 17 patients with GBM, 1 with gliosarcoma, 1 with anaplastic oligodendroglioma and 1 patient with spindle cell metastasis) through an intracerebral convection-enhanced delivery (CED) technique has been conducted. This study closed prematurely during the dose escalation phase due to inconsistent drug delivery. The median survival of the overall population was 28 weeks (95% CI, 26.5–102.8), while of 15 patients with residual measurable disease, 2 (13.3%) had radiographic responses, including 1 GBM patient, who had a nearly complete response (CR) [23].

Beyond EGFR, other targets being investigated included interleukin-13 (IL-13), interleukin-4 (IL-4) and the transferrin receptor (Table 2).

A randomized phase III study compared the efficacy of Cintredekin besudotox (CB), an immunotoxin composed of IL-13 and an engineered truncated form of PE delivered intracranially through CED, with Gliadel wafers as a form of treatment of GBM at first recurrence, after a salvage surgical resection. No survival difference was observed between the two treatment arms [28] and the safety profiles were comparable, except for the number of pulmonary embolisms, which was higher in patients treated with CB.

MDNA55 (Bizaxofusp), an IL-4 receptor-targeting toxin administered intratumorally via CED, has been investigated as a single agent in a single-arm phase IIb study including 47 patients with recurrent GBM (rGBM). MDNA55 showed an acceptable safety profile at doses up to 240 μg. The overall tumor control rate evaluated according to modified Response Assessment in Neuro-Oncology (RANO) criteria, including tumor shrinkage or stabilization, was 75.6% (31 of 41 patients), with 1 patient exhibiting a durable CR following initial radiographic changes consistent with pseudoprogression. The most significant benefit has been observed in the subgroup of IL4R High + IL4R Low^HD^ patients with a TCR of 81%. The median overall survival (mOS) and OS at 12 months were 11.64 months (80% one-sided CI 8.62, 15.02) and 46%, respectively [29]. Based on these results, it has been since granted Fast Track (Food and Drug Administration—FDA) and orphan drug status (FDA and European Medicines Agency—EMA) for the treatment of rGBM [30].

Tf-CRM107 is an immunotoxin carrying a modified DT payload covalently bound to transferrin [31]. Two phase III trials of Tf-CRM107 were initiated, but unfortunately, one was withdrawn following a probability analysis performed prior to the recruitment of patients, determining that it was unlikely that the agent would meet FDA criteria for efficacy (NCT00083447; clinicaltrials.gov, Accessed on 5 May 2025). The other one instead compared Tf-CRM107 with standard treatment. Despite the accrual having been completed, results were never released (NCT00088400; clinicaltrials.gov, Accessed on 5 May 2025).

More recently, the ADC that raised the greatest expectations for GBM treatment was Depatux-M. This agent comprises an anti-EGFRvIII mAb (depatuximab) connected via a non-cleavable linker to a microtubule inhibitor, monomethylauristatin-F (MMAF). The mAb depatuximab not only targets the EGFRvIII, but is also able to bind to wild-type EGFR on neoplastic cells with EGFR amplification, even in the absence of EGFRvIII co-mutation, by binding to a tumor-specific, conformationally exposed epitope [32].

The safety, pharmacokinetics (PK), and preliminary antitumor activity of Depatux-M, given either alone in rGBM or in combination with temozolomide (TMZ) in newly diagnosed (nGBM) or rGBM, were tested in a large multicenter, three-arm, phase I trial. Arm A included patients with nGBM who were to receive Depatux-M + RT + TMZ; arm B included both newly diagnosed or recurrent GBM patients who were to receive Depatux-M + TMZ after RT; and arm C included enrolled patients with rGBM who were to receive Depatux-M in monotherapy. Each arm was composed of a dose escalation and dose expansion cohort. The most common adverse events (AEs) were ocular, occurring in 87% of patients, including blurred vision (63%) and photophobia (35%), while the most common non-ocular AEs were thrombocytopenia (45%, attributable to TMZ, potentially exacerbated by concurrent Depatux-m) and fatigue (38%). Dose delays occurred in 35 patients (58%), most commonly due to ocular side effects, while no treatment discontinuations due to ocular side effects were reported. PK exposures of Depatux-M, total Depatux, and cys-mafodotin after i.v. infusion of Depatux-M were approximately dose-proportional over the dose range of 0.5–1.5 mg/kg. Coadministration of Depatux-M did not affect the PK parameters of TMZ. Among 58 patients with RANO-defined measurable disease at baseline, the overall response rate (ORR) was 13.8% (1/58 CR, 7/58 PR, 95% CI = 6.2%, 25.4%), 26 patients had stable disease (SD), and 24 patients had progressive disease (PD). The median duration of response (mDOR) was 5.6 months (95% CI = 1.5, 9.7). The median progression-free survival (mPFS) and mOS of the entire study population were 2.1 months (95% CI = 1.1, 3.4) and 7.4 months (95% CI = 6.5, 9.6), respectively.

Based on this acceptable safety profile and the preliminary efficacy results, Depatux-M [33,34,35,36] was further tested in larger phase II and III trials.

**Table 2:** Trials on immunotoxins with targets other than EGFR in GBM.

Reference	Type of Trial	Population	Intervention	Target	Endpoints
[35]	Phase III	rGBM (first recurrence)	CB vs. gliadel wafers	IL-13	No survival difference
[36]	Phase IIb	rGBM	MDNA55 (Bizaxofusp)	IL-4	mOS: 11.64 months [80% one-sided CI 8.62, 15.02]

Abbreviations: CB, Cintredekin besudotox; rGBM, recurrent GBM.

In the randomized phase II study INTELLANCE-2, 260 patients with EGFR-amplified rGBM were randomized to receive Depatux-M in combination with TMZ (ARM 1), Depatux-M alone (ARM 2), TMZ or lomustine (ARM 3). At a median follow-up of 28.7 months, a significant benefit in OS was observed in the combination arm vs. control arm 3 (HR 0.66, 95% CI 0.47–0.93; *p* = 0.017). Conversely, no significant difference was observed between the Depatux-M monotherapy and the control arm (HR 0.96, 95% CI 0.69–1.33; *p* = 0.80). The 2-year survival was 19.8% (95% CI 12.2, 28.8), 10% (95% CI 4.8, 17.6), and 5.2% (95% CI 1.7, 11.7), in the combination arm, Depatux-M monotherapy arm, and control arm, respectively. No significant differences according to the O6-Methylguanine-DNA methyltransferase (MGMT) promoter methylation status were observed, neither in the monotherapy nor in the combination arms. A trend toward increased clinical benefit from the combination of Depatux-M + TMZ was seen in patients relapsing > 16 weeks after the end of TMZ treatment, with a 2-year survival of 28.6% (95% CI 13.5, 45.6) for the combination arm, vs. 11.1% (95% CI 2.8, 25.9) for the Depatux-M monotherapy arm, and 3.9% (95% CI 0.3, 16.4) for the control arm [37].

The subsequent INTELLANCE-1 study was a large randomized, placebo-controlled phase III trial designed to compare the addition of Depatux-M to chemo-radiation vs. the standard Stupp regimen, in patients with EGFR-amplified nGBM, with OS as the primary endpoint. The trial did not meet its primary endpoint, showing no OS benefit for the experimental arm with an mOS of 18.9 vs. 18.7 months in the control arm (HR 1.02, 95% CI 0.82–1.26; 1-sided *p* = 0.63). Progression-free survival (PFS) was longer for patients who received Depatux-M compared to those who received a placebo, with an mPFS of 8.0 vs. 6.3 months (HR 0.84, 95% CI 0.70–1.01; *p* = 0.029). This benefit was more relevant in patients harboring the EGFRvIII mutation (mPFS 8.3 vs. 5.9 months, HR 0.72, 95% CI 0.56–0.93; 1-sided *p* = 0.002) and for MGMT-unmethylated patients (HR 0.77, 95% CI 0.61–0.97; 1-sided *p* = 0.012). These data raised the question of whether the EGFR-vIII mutation might represent a better selection biomarker for trial eligibility rather than amplification. The enrolled population in this case included only approximately 50% of EGFRvIII-mutated patients. However, it is important to underscore that high intratumor heterogeneity significantly impacts the predictive value of both EGFRvIII and receptor amplification [38]. Corneal epitheliopathy was confirmed as the most common treatment-related adverse event (TRAE) occurring in 94% of patients of the experimental arm, of which 61% were grade 3–4, leading to a 12% discontinuation rate.

Given the failure to meet the primary endpoint, this study was discontinued early and unblinded for futility [39].

A real-world study of Depatux-M + TMZ in rGBM was conducted by an Italian group and demonstrated results comparable with those of the phase II trial INTELLANCE-2, finding that the only factor significantly associated with a better outcome was the MGMT methylation status [40].

Another transmembrane protein, the human trophoblast cell surface antigen 2 (TROP2), was recently correlated with GBM proliferation capacity [41]. Interestingly, its amplification was associated with its promoter hypomethylation, albeit in a very small sample [42,43].

Moreover, normal human brain tissue does not express TROP2, potentially increasing the therapeutic index of ADCs in this setting [44]. A phase II multicenter, prospective study of SG in rGBM has now entered the interim analyses [41].

Currently ongoing trials assessing the efficacy of ADCs in primary CNS tumors are listed in Table 3.

## 3. Brain Metastases

### 3.1. Non-Small Cell Lung Cancer

Approximately 50% of patients with advanced non-small cell lung cancer (NSCLC) develop BMs throughout the course of their disease. The incidence of BMs is particularly high in NSCLC, with EGFR mutations or anaplastic lymphoma kinase (ALK) rearrangements with a baseline occurrence around of 30–35% in both groups [45,46].

The emergence of BMs represents a relevant challenge in the clinical management of these patients, given their association with poor prognosis, neurological deterioration, and reduced quality of life.

As underlined above, the historical management of BMs, relying on RT and surgery, has been recently challenged by the advent of new anticancer agents, such as ICIs and TKIs targeting actionable genetic alterations. This is particularly true in the context of lung cancer, with the entry into the market of several compounds which have proven to be truly active in the CNS, leading to excellent intracranial disease control [47]; this is the case for EGFR and ALK inhibitors or anti PD-(L)1 monoclonal antibodies (mABs).

In a subgroup analysis of the phase III FLAURA trial, osimertinib, a third-generation EGFR TKI, compared to erlotinib or gefitinib in a population of treatment-naïve, EGFRm NSCLC patients, was associated with a 52% reduction in the risk of CNS progression or death due to its superior ability to cross the BBB [48]. The great ability of osimertinib in controlling intracranial disease was further confirmed in the phase III FLAURA 2, with an intracranial objective response rate (iORR) of 73% being reached in the chemotherapy combination arm and 69% in the monotherapy arm [49].

In a population of treatment-naïve ALK-positive NSCLC patients with BMs enrolled in a phase III CROWN study, the third-generation ALK TKI lorlatinib led to an impressive iORR, assessed by a RECIST v1.1 value of 60%, with an intracranial complete response rate (iCRR) of 49% [50].

In non-oncogene-addicted metastatic NSCLC, a pooled analysis of the KEYNOTE-021, KEYNOTE-189, and KEYNOTE-407 studies, which included patients with stable BMs at baseline, showed that the addition of pembrolizumab significantly improved OS compared to chemotherapy alone. Unfortunately, iORRs are not available since the lesions were considered to be non-targets within the studies [51].

In the ATEZO-Brain [52] trial, the combination of anti PD-L1 Atezolizumab + chemotherapy in a population of NSCLC patients and untreated BM achieved an iORR of 42.7%, assessed with RANO and RECIST 1.1 criteria. In the CAP-BRAIN [53] trial, Camrelizumab + chemotherapy was evaluated in a population, with more than three MRI-confirmed BMs or one to two BMs not amenable for local therapy. Camrelizumab plus chemotherapy led to an iORR of 46.7%, as assessed with RECIST 1.1 criteria.

In this quickly evolving landscape, ADCs are emerging as a new therapeutic strategy for the treatment of NSCLC, and understanding their degree of CNS activity is crucial to determining if they represent a novel option even in the presence of BMs, particularly when RT is not feasible, chemo-immunotherapy fails and resistance to targeted drugs occurs [54].

#### 3.1.1. Trastuzumab Deruxtecan

To date, the first and only FDA-approved ADC for the treatment of lung cancer is trastuzumab deruxtecan (T-DXd). T-DXd consists of an antibody targeting the human epidermal growth factor receptor 2 (HER2) linked to a topoisomerase I inhibitor, cytotoxic payload deruxtecan (DX-d), via a cleavable linker. HER2 is a transmembrane receptor of the EGFR family that activates through dimerization, promoting HER2 pathway activation. In NSCLC, up to 20% of patients have HER2 overexpression while only 2% bear a targetable mutation. These mutations are commonly linked to adenocarcinoma histology and are more frequently observed in females, individuals of Asian descent, and those who have never smoked [55].

In August 2022, the FDA granted accelerated approval of T-DXd for the treatment of unresectable/metastatic NSCLC patients with an HER2 mutation, based on results of the DESTINY-Lung01 and DESTINY-Lung02 trials.

The DESTINY-Lung01 and DESTINY-Lung02 trials were multicenter, single-arm, phase II studies evaluating the efficacy of T-DXd in patients with unresectable or metastatic NSCLC carrying HER2 mutations or overexpression. DESTINY-Lung01 enrolled a total of 181 patients; 91 patients were enrolled in cohort 2 (HER2 mutated cohort) and treated with T-DXd at a dose of 6.4 mg/kg every 3 weeks, while 90 patients were enrolled in cohort 1 (HER2 overexpressing cohort) and treated with an either 5.4 mg/Kg (cohort 1) or 6.4 mg/kg dose (cohort 1a). DESTINY-Lung02 enrolled 152 patients with HER2 mutation who were randomized to receive either T-DXd at a dose of 5.4 mg/kg or 6.4 mg/kg every 3 weeks. Patients with stable (asymptomatic and not requiring steroid treatment) BMs were allowed to participate in both studies. In the pooled analyses from DESTINY-Lung01 and DESTINY-Lung02 trials, T-DXd showed excellent intracranial activity. In the 5.4 mg/kg treatment cohort, the iORR, according to RECIST v1.1, was 25% (8/32), with four iCRs and four intracranial partial responses (iPRs), while an intracranial stable disease (iSD) was observed in 56.3% (18/32) of cases. In the 6.4 mg/kg cohort, 10 out of 54 (18.5%) patients had a response to T-DXd, including 9 iPRs and 1 iCRs, while 31 had patients had an iSD (57.4%) [56].

Nevertheless, many ADCs are currently under development for the treatment of NSCLC. In this section, we will review the key ADCs being investigated and those for which intracranial response data are available (Table 4).

#### 3.1.2. Datopotamab Deruxtecan

Datopotamab deruxtecan (Dato-DXd) is an ADC that targets the TROP2 antigen, linked to the topoisomerase I inhibitor deruxtecan via a cleavable linker. Trop2 is a type-1 surface glycoprotein that binds to various partners such as claudins 1 and 7, cyclin D1, and insulin-like growth factor 1 (IGF-1) [57]. It plays an important role in stabilizing epithelial tight junctions and regulating cell growth and proliferation, and is overexpressed in approximately 64% of adenocarcinomas and up to 75% of squamous cell carcinomas [58,59]. In the single-arm phase II TROPION-Lung05 trial, Dato-DXd was investigated as a treatment for patients with metastatic NSCLC and at least one or more genomic alterations, including EGFR, ALK, ROS proto oncogene 1 (ROS1), neurotrophic tyrosine receptor kinase (NTRK), b-raf proto-oncogene (BRAF), rearranged during transfection (RET), or mesenchymal–epithelial transition factor (MET) exon 14 skipping, who received up to four prior lines of treatment, including at least one target therapy. Patients with BMs were eligible if they were asymptomatic without steroid therapy.

Data on intracranial activity from a prespecified exploratory analysis were presented at the 2024 ASCO annual meeting. Among the 53 patients with BMs, treatment with Dato-DXd led to an intriguing iORR of 22%, as assessed by RECIST v1.1, along with an intracranial progression-free survival (iPFS) of 6.9 months. The ORR was 28% in patients with baseline BMs and 40% in those without.

#### 3.1.3. Patritumab Deruxtecan

Patritumab deruxtecan (HER3-DXd) is an ADC that targets the human epidermal growth factor 3 (HER3) antigen, linked to the topoisomerase I inhibitor DXd via a cleavable linker. HER3 is often overexpressed in many tumors, including NSCLC, with a prevalence of 83% in primary tumors. Binding to its ligand, heregulin, HER-3 increases tumor proliferation and metastatic capacity. Recently, the role of HER3 as a resistance factor to TKIs has emerged. Dimerization with other receptors such as HER2, EGFR, and MET leads to proliferation signals that may cause cells to resist targeted therapy [60,61]. HER3-Dxd has been evaluated in the phase II HERTHENA-Lung01 (NCT04619004) trial in participants with metastatic or locally advanced NSCLC with canonical activating EGFR mutations (exon 19 deletion or L858R) who have received and progressed on or after at least one EGFR TKI and one platinum-based chemotherapy-containing regimen. Patients with baseline BMs were eligible if clinically silent with a need of daily prednisone <10 mg/day or equivalent. Out of 225 total patients, 30 had non-irradiated BM at baseline. In this subgroup, HER3-DXd demonstrated an iORR of 33.3% as assessed by Blinded Independent Central Review (BICR) per RECIST criteria.

#### 3.1.4. Telisotuzumab–Vedotin

Telisotuzumab–Vedotin (Teliso-V) is a c-MET-directed ADC with a monomethyl auristatin E cytotoxic payload. Met is a proto-oncogene that is activated by hepatocyte growth factor (HGF). Its hyperactivation in NSCLC is mediated by several mechanisms such as amplification and skipping of exon 14 [62].

The Phase 2 LUMINOSITY trial evaluated Teliso-V in locally advanced/metastatic non-squamous NSCLC patients with MET overexpression. Patients with BMs were eligible if the metastases received definitive treatment and were stable. Out of the 161 NSCLC patients included in the study, 33 (20.5%) had stable and previously treated BMs at the study entry. The trial demonstrated an ORR of 34.6% in c-Met high patients, 22.6% in c-Met intermediate patients and 28.6% in the intention to treat (ITT) population. Unfortunately, subgroup analyses regarding intracranial activity have not yet been presented [63].

Several other ADCs targeting molecules such as EGFR, folate receptor 1 (FOLR1), receptor tyrosine kinase-like orphan receptor 2 (ROR2) and AXL receptor tyrosine kinase (AXL) are currently under investigation for the treatment of NSCLC, but data on intracranial activity are not yet available (Table A1).

### 3.2. Breast Cancer

Breast cancer (BC) is the second most common malignancy causing BMs, after lung cancer [64]. Among the BC molecular subgroups, HER2-positive and triple-negative breast cancer (TNBC) are those most frequently associated with the development of BMs, with a cumulative incidence of 31% and 32%, respectively, while CNS involvement is rarer in the luminal subtype, with a cumulative incidence of 15% [65].

The prognosis of patients with BC and BMs remains quite poor, with an mOS of 8.7 months, but there are significant differences according to the molecular subtypes, ranging from 9.3, 16.5, and 21.6 to 4.9 months in the luminal, HER2-positive and TNBC group, respectively [66].

Recently, Chehade et al. demonstrated that most surgically resected BMs can be classified as HER-2-low by immunohistochemistry (IHC), thus potentially being druggable with HER-2-directed ADCs [67]. Another potential target was highlighted in a phase 0 trial, where patients with breast cancer brain metastasis (BCBM) received SG before surgery. In this study, 100% of TNBC BM tumor samples demonstrated TROP2 expression with a 3+ score for IHC [68].

In this section, we will review FDA-approved ADCs for treating BC, for which intracranial response data are available, including Trastuzumab emtansine (T-DM1), T-DXd and Sacituzumab Govitecan (SG) (Table 5).

T-DM1 comprises the mAb trastuzumab conjugated via a non-cleavable linker to the potent tubulin polymerisation inhibitor mertansine (DM1). It is indicated in the adjuvant treatment of patients with HER2-positive early breast cancer with residual invasive disease after the administration of a trastuzumab- and taxane-based neoadjuvant regimen and in the advanced setting, for patients with HER2-positive MBC who previously received trastuzumab and a taxane, separately or in combination.

T-DXd is indicated for the treatment of HER2-positive advanced breast cancer (aBC), previously treated with an anti-HER2-based regimen in the metastatic/neoadjuvant/adjuvant setting, if disease recurrence occurs during or within 6 months of completing therapy. In August 2022, the FDA approved T-DXd also for the treatment of patients with unresectable or metastatic HER2-low (immunohistochemistry [IHC] IHC 1+ or IHC 2+ with a negative in situ hybridization test [ISH−]) breast cancer, who had received prior chemotherapy in the metastatic setting or developed disease recurrence during or within 6 months of completing adjuvant chemotherapy.

The third approved ADC in the context of aBC is SG, a conjugate composed of an antibody targeting the antigen TROP2, which is expressed in most breast cancers, coupled to SN-38 (topoisomerase I inhibitor) through a hydrolyzable linker. SG was approved by the FDA on February 2023 for patients with unresectable, locally advanced or metastatic hormone receptor (HR)-positive, HER2-negative (IHC 0, IHC 1+, or IHC 2+ with ISH−]) breast cancer who have received endocrine-based therapy and at least two additional systemic therapies in the metastatic setting.

#### 3.2.1. Ado-Trastuzumab Emtansine

In the (neo)adjuvant setting, T-DM1 did not demonstrate a decrease in the risk of BMs, probably because of its scarce penetration into an intact BBB [69,70].

The randomized phase III EMILIA trial established T-DM1 as the standard second-line treatment for patients with HER2-positive aBC previously treated with trastuzumab and a taxane, showing superiority over lapatinib + capecitabine (XL) in terms of PFS and OS (mOS of 29.9 months (95% CI 26.3–34.1) vs. 25.9 (95% CI 22.7–28.3); HR 0.75 (95% CI 0.64–0.88) [71].

A retrospective exploratory analysis of this trial assessed the incidence of BMs after treatment with T-DM1 vs. XL and treatment efficacy among patients with pre-existing CNS metastases. Considering patients with no BMs at baseline, CNS progression occurred in 9 of 450 (2%) in the T-DM1 and in 3 of 446 (0.7%) in the XL arms. Among the 95 patients with previously treated and asymptomatic BMs at baseline, T-DM1 (n = 45) has been demonstrated to be superior to XL in prolonging intracranial disease control in this specific population, with a mPFS of 5.9 months (vs 5.7 in the control arm) (HR 1.00; 95% CI 0.54–1.84; *p* = 1.000), and an mOS of 26.8 (vs 12.9 months in the control arm) (HR 0.38, 95% CI 0.18–0.79; *p* = 0.0081) [72].

In the randomized, open-label, phase III TH3RESA trial, T-DM1 was compared to the treatment of physician’s choice (TPC) for patients with metastatic breast cancer (mBC) pretreated with ≥2 HER2-directed regimens. The systemic mPFS was 6.2 vs. 3.3 months in the T-DM1 group vs. the control arm, respectively (HR 0.53, 95% CI 0.422–0.661; *p* < 0.0001). Considering only patients with asymptomatic, previously treated CNS metastases, the mPFS was 5.8 months in the T-DM1 (n = 40) arm vs. 2.9 months in the control arm (n = 27) (HR 0.47, 95% CI; 0.24–0.89) [73].

The single-arm, open-label phase IIIb KAMILLA trial [74] tested T-DM1 in 2002 patients with HER2-positive locally a/mBC treated with prior HER2-targeted therapy and chemotherapy, of which 398 had BMs (controlled or untreated and asymptomatic). Tumor response and clinical outcomes in patients with baseline BMs were evaluated in a post hoc exploratory analysis. Among the 126 cases with measurable BMs, according to RECIST v1.1, the iORR was 21.4%, with an iCR of 2.4%. The mPFS was 5.5 months (95% CI 5.3–5.6) and the mOS was 18.9 months (95% CI 17.1–21.3). A reduction of 30% or more in the sum of the largest diameters of target brain lesions was observed in 50.0% (5/10; 95% CI 18.7–81.3) and 32.7% (16/49; 95% CI 20.0–47.6) of patients treated with brain RT < 30 days and ≥30 days before study entry, respectively, and in 49.3% of those who did not receive brain RT (33/67; 95% CI 36.9–61.8).

#### 3.2.2. Trastuzumab Deruxtecan

Another agent recently introduced among the therapeutic armamentarium of HER2-positive mBC is T-DXd.

In the phase II DESTINY-Breast01 trial, 24 patients with previously treated and asymptomatic BMs were included. The iORR, according to RECIST v1.1 criteria, of the 17 patients with measurable BMs at baseline was 41.2% [75,76], while the mPFS of the entire BM cohort was 18.1 months (95% CI 6.7–18.1).

The phase III DESTINY-Breast03 trial evaluated the efficacy and safety of T-DXd vs. T-DM1 in HER2-positive mBC patients who progressed to trastuzumab and a taxane. In the 82 patients with inactive and/or previously treated BMs enrolled in this trial, T-DXd showed a significantly improved iORR, according to RECIST v1.1 criteria, compared to T-DM1 (79 vs. 35%, respectively) [19]. The considerable CNS activity of T-DXd was then confirmed in the TUXEDO-1 trial, a prospective, single-arm, phase 2 study recruiting patients with HER2-positive BC and untreated BMs or BMs progressing after previous local therapy, after previous exposure to trastuzumab and pertuzumab and with no indication for immediate local therapy. Among the 15 patients enrolled in the ITT population, the iORR was 73.3% (95% CI 48.1–89.1%), meeting the predefined primary endpoint, with 2 patients (13.3%) experiencing an iCR, 9 (60%) an iPR and 3 (20%) iSD [77].

The largest amount of prospective data of T-DXd in patients with BMs so far have come from the DESTINY-Breast12 trial [78]. This is a multicenter, phase IIIb/IV, open-label, two-cohort study of T-DXd in patients with previously treated HER2-positive mBC with and without BMs. Of the 504 patients enrolled in the trial, 263 had BMs, of which 157 were previously treated and considered stable, while 106 were active (39 untreated and 67 previously treated BMs progressive at time of study entry—without indication for immediate retreatment with local therapy). T-DXd demonstrated substantial and durable intracranial clinical activity, with an overall CNS response rate of 71.7% within the 138 cases with baseline measurable CNS disease (stable BMs: n = 77; active BMs: n = 61). The iORR was 79.2% (95% CI 70.2–88.3) and 62.3% (95% CI 50.1–74.5) in patients with stable and active BMs, respectively. Interestingly, the 12-month survival rate of patients with and without BMs was comparable, at 90.6% and 90.3%, respectively. The safety profile of T-DXd was consistent with that in previous reports, while interstitial lung disease/pneumonitis was confirmed as an important safety risk of T-DXd, with six related deaths in the BM cohort, five of which occurred in patients taking steroids.

Another example of strong confirmation of T-DXd CNS activity can be found in the ongoing phase II DEBBRAH trial. This study evaluates patients with pretreated HER2-positive or HER2-low advanced BC with stable, untreated, or progressing BMs, and/or leptomeningeal carcinomatosis, allocated into five cohorts based on HER2 expression and metastatic brain status. Cohort 1 comprises patients with non-progressing BMs after local therapy (n = 8), cohort 2 includes asymptomatic untreated BMs (n = 4), and cohort 3 includes those with progressing BMs after local therapy (n = 9). Overall, the iORR in patients with active BMs was 46.2% (95% CI, 19.2–74.9), 50.0% (95% CI, 6.7–93.2) in cohort 2 and 44.4% (95% CI, 13.7–78.8; *p* < 0.001) in cohort 3, respectively. In cohort 1, the 16-week PFS rate was 87.5% (95% CI, 47.3–99.7; *p* < 0.001) [79].

Currently, the evidence on HER2-low BCBM is limited. A deeper understanding of the clinicopathological characteristics and prognosis of HER2-low BCBM is essential. The results attained by T-DXd in HER-2 low and HER-2 ultra-low subtypes leverage a definition as a spectrum continuum of expression of HER-2 in mBC [80]. The phase 2 DAISY trial evaluated the T-DXd response rate (RR) based on HER-2 expression, including those with treated and asymptomatic BM [81,82]. Results showed an ORR of 70%, 36%, and 30% for HER-2 positive (3+ by IHC), HER-2 low (1+ or 2+ by IHC, ISH negative), and HER-2 negative (0+ by IHC) BC cohorts, respectively. Similarly, in the DESTINY-Breast04 trial, 35 patients with stable and treated BMs showed improved intracranial responses with T-DXd in HER2-low disease compared to the TPC [83]. Results from cohorts 2 and 4 of the DEBBRAH trial evaluating T-DXd in patients with active HER2-low BMs showed an iORR per RANO-BM of 50.0% in patients with asymptomatic untreated BMs (n = 6, cohort 2) and 33.3% in those with progressing BMs (n = 6, cohort 4), with a median duration of intracranial response of 7.2 months [84]. Despite being promising, the performance of T-DXd in active BMs was assessed in very small populations, so studies enrolling larger populations are needed.

#### 3.2.3. Sacituzumab Govitecan

The randomized phase III ASCENT trial investigated the efficacy of SG compared with that of single-agent TPC (eribulin, vinorelbine, capecitabine, or gemcitabine) in patients with relapsed or refractory metastatic triple-negative breast cancer (mTNBC) [85]. In this trial, patients with stable BMs were eligible but not included in the primary endpoint analysis; these patients may constitute a maximum of 15% of the overall trial population. In the subgroup analysis of the 61 patients with BMs at study entry, a trend toward improvement in mPFS was demonstrated in favor of SG vs. TPC (2.8 months vs. 1.6 months; HR 0.68; 95% CI 0.38–1.23), however without any benefits for OS (7.0 vs. 7.5 months, respectively; HR 0.96; 95% CI 0.55–1.68) [86].

Some of the most relevant ongoing trials on ADC in mBC are listed in Table 6. Data on intracranial efficacy are not available as of today.

## 4. Combination with Radiation Therapy

Given the relatively recent introduction of ADCs in clinical practice, there is a current lack of knowledge about the potential synergy with other anticancer treatment strategies, such as RT.

The concomitant use of ADCs and RT has not been assessed in prospective clinical trials so far. Historically, the rationale for combining chemotherapy and RT stems, among other mechanisms, from the ability of some chemotherapeutic agents to block the cell cycle in the M, late G1 and S phases, in which cells are most susceptible to radiation damage (5-FU, gemcitabine) [87].

Given the mechanism of action of ADCs, it seems reasonable that the same effects could be obtained by the toxic payloads combined with RT. Most of the scarce data available come from BC, in particular from the use of T-DM1, the first ADC approved for breast cancer. No increased toxicity was reported with the concomitant administration of T-DM1 and whole-brain RT (WBRT) in case reports and preclinical cellular models [88,89,90,91].

In contrast, the use of T-DM1 concomitant to stereotactic radiosurgery SRS seems to come with an increased risk of neurological side effects, including clinically significant radionecrosis expansive hematoma in delayed cerebral radiation necrosis [92,93], intracranial hemorrhages [94], and brain edema [95,96].

One possible mechanism underlying this increased toxicity is an off-tumor on-target effect, driven by the expression of HER2 on astrocytes [70,97], which may be targeted by ADCs, with the release of their cytotoxic payloads.

In a large retrospective cohort study conducted by Lebow et al., data on 98 patients with BMs from different cancer types (NSCLC, BC, HER2 amplified Esophageal and/or gastric cancer, HER2 amplified Salivary) who received at least one course of stereotactic RT (SRT) concurrently with T-DM1, T-DXd, or SG were reviewed. SRS was considered concurrent if it was delivered 7 or fewer days before or 21 or fewer days after ADC, and a control group of patients treated with SRS and the ADC sequentially was created for comparison. The concurrent administration of ADC with SRS was associated with a higher risk of symptomatic radionecrosis, both on univariable analysis (SHR, 4.01 [95% CI, 1.79–9.01]; *p* < 0.001) and multivariable analysis (SHR, 4.31 [95% CI, 1.95–9.50]; *p* < 0.001), controlling for prior RT (SHR, 2.99 [95% CI, 1.26–7.09]; *p* = 0.01) and BM volume (SHR, 1.14/cm^3^ [95% CI, 1.09–1.19]/cm^3^; *p* < 0.001). This risk was significantly more pronounced for larger and previously irradiated BMs [98].

Among the concurrent treatment group, the specific ADC was not associated with a risk of radionecrosis (*p* = 0.74). Given the high rates of local control and its favorable neurocognitive safety profile, SRS remains a key therapeutic option in BMs. However, physicians must consider the increased risk of radionecrosis and monitor patients closely when treating them concurrently with ADCs, or avoid the concomitant use of SRS and ADCs if possible.

## 5. Discussion

This review aims to summarize the current evidence on ADC therapies in the specific context of the CNS when used against BMs or adult-type gliomas.

Despite their well-recognized lack of systemic therapeutic options, these two populations have historically been underrepresented in clinical trials [99]. The poor prognosis of these patients, coupled with their frequent rapid clinical deterioration and the mechanisms of resistance to most therapeutic strategies, likely explains why patients with symptomatic or active BMs have traditionally been excluded from large, randomized trials. However, their underrepresentation, particularly in early-phase clinical trials, is difficult to justify, as these patients could play a valuable role in assessing the safety and determining the recommended doses of experimental drugs, providing valuable information about the activity of novel compounds at the CNS level [100].

Meanwhile, ADCs have emerged as a new effective class of anticancer agents currently revolutionizing the treatment paradigm of a wide range of different tumor types, attaining seven FDA approvals so far, with numerous trials still underway [101]. Given the very recent advent of ADCs in the anticancer therapeutic armamentarium, most of the data covered in this review are retrospective or derived from post hoc analyses of prospective trials. They are focused on a limited number of cancer histotypes, predominantly NSCLC and BC.

Over the years, the continuous technological evolution of ADCs has seen the development of these new drugs up to their third generation. T-DXd is considered the first exemplary drug of the third-generation anti-HER2 ADCs because of its technological advancements in the linker, DAR, and payload. Its linker allows the conjugation of up to eight cytotoxic payload molecules (DX-d) while not reducing the compound’s half-life [102]. Together with the high membrane permeability of the DX-d compound, it explains the higher intracranial activity of T-DXd compared to that of its second-generation predecessor, T-DM1, in HER2-positive BCBM, with encouraging preliminary results in HER2-low BM tumors [103].

It is more complex to compare the intracranial activity of SG with that of T-DXd in two different subtypes of BC, TNBC and HER2-positive. Both drugs possess the typical characteristics of third-generation ADCs, such as high DAR, cleavable linker, and bystander effect. One possible explanation for the higher T-DXd activity is that it targets an oncogenic driver, whereas SG is directed against a transmembrane protein frequently overexpressed across various carcinomas. However, this contrasts with the promising intracranial results observed in the HERTHENA-Lung01 trial using HER3-DXd in EGFR-mutant NSCLC, where HER3 is unlikely to act as the primary oncogenic driver. This discrepancy highlights the still-unknown mechanisms underlying the variability in intracranial responses to different ADCs.

Intricate is the challenge in gliomas headed by GBM, the most aggressive primary brain malignancy, which has not yet seen any ADC-based therapy approval. We previously underscored the heterogeneity of EGFR amplification and its mutated form EGFRvIII in GBM cells, which could potentially lead to the failure of INTELLANCE-1 trial. Furthermore, Depatux-M requires complete lysosomal degradation to function, as it does not have a cleavable linker, and the MMAF payload hardly crosses intact cell membranes [104]. These technical features may have resulted in the early selection of resistant clones and justified the loss of survival advantage. In contrast to BMs, the diffuse infiltrative nature of glioblastoma cells, especially in the peripheral area where the BBB may retain its physiological functions, could pose a substantial hurdle to ADC efficacy.

One potential strategy is to move away from the traditional mAb structure and couple the payload to a polypeptide or single-chain variable fragment with a smaller molecular weight, enhancing penetration efficiency and payload delivery to tumor tissues [105]. Another possible approach proposed in a study led by Anami et al. involves optimizing ADCs with homogeneous DAR values, which were shown to achieve higher brain bioavailability. High-DAR components in heterogeneous ADCs demonstrate poor CNS penetration, leading to reduced effective DAR compared to that of their homogeneous counterpart [106]. They prove that ADCs with high DAR have greater hydrophobicity, form macromolecules and are characterized by reduced plasma stability, leading to impaired BTB penetration and increased off-target toxicity [107].

Traditional ADCs that utilize cysteine or lysine amino acids as attachment points exhibit significant variability in DAR. In contrast, site-specific conjugation techniques, such as THIOMAB, can lead to the generation of homogeneous ADCs characterized by a constant DAR, by exploiting the insertion of cysteine residues at different positions at the light and heavy chains for conjugation [108]. Several other conjugation techniques are being investigated, including the use of microbial transglutaminase, an enzyme that normally catalyzes post-translational modifications of proteins, which can facilitate the conjugation process and provide flexibility in the selection of the conjugation site and DAR [109]. Another promising technique is AJICAP-M, a traceless affinity peptide-mediated conjugation strategy, which can produce more stable ADCs with minimized payload detachment, suggesting a higher therapeutic index than that of conventional ADCs [110].

For this reason, manufacturing homogeneous ADCs with optimized DAR can potentially increase payload delivery in pBTs and BMs.

The bystander effect also appears to be important in determining the activity of ADCs at the CNS level by allowing cytotoxic activity even against target-negative cells. The choice of a payload that is small, lipophilic and that is not a substrate of the P-gp could be a strategy to allow a better penetration at the CNS level. This could in part explain the higher activity of T-DXd compared to that of T-DM1, the payload of the latter being a substrate of the efflux systems of the BBB [111].

Besides the payload, the type and stability of the linker are of paramount importance.

New techniques including exo-linkers, with their two-step activation system, and tandem linkers incorporating several cleavage steps have the potential to increase plasma stability and reduce premature payload release, potentially reducing systemic toxicities and enabling better payload distribution within the CNS [112,113,114].

Leptomeningeal disease is still an entity excluded from most prospective clinical trials, and current systemic treatment approaches are derived from BMs. In cohort 5 of the DEBBRAH trial, T-DXd was tested on a total of 7 patients with untreated leptomeningeal involvement, leading to an mOS of 13.3 months and an mPFS of 11.9 months. Of the five patients who progressed, none of them had intracranial progression or worsening of symptoms referable to leptomeningeal carcinomatosis. Although these data are from a very small number of patients and should be confirmed by further larger prospective studies, they provide an early indication of the efficacy of ADCs in the treatment of leptomeningeal carcinomatosis [115].

At the methodological level, the prospective TUXEDO-1 and DEBBRAH trials currently constitute the most rigorous and statistically appropriate frameworks for the dedicated evaluation of intracranial activity-associated ADCs. In TUXEDO-1, baseline patient characteristics are comprehensively delineated, with particular attention to neurological symptomatology, prior local therapeutic interventions, and the dynamic status of intracranial disease, distinguishing between progressive and newly diagnosed lesions. The DEBBRAH trial adopts an even more granular stratification strategy, enrolling patients into five discrete cohorts according to HER2 membrane receptor expression, previous intracranial treatment history, the presence or absence of neurological symptoms, and leptomeningeal dissemination. Such meticulous cohorting facilitates a highly refined interrogation of ADC efficacy within clinically heterogeneous populations, addressing variables often overlooked in broader trial designs. The remaining studies have some critical issues as they were not formally designed to study the intracranial activity of ADCs. While approximately 75% of studies report including CNS-specific endpoints, only a minority, one-third, permit the enrollment of patients with active or progressing intracranial disease.

Another key issue highlighted by the studies collected in this review and limiting the ability to analyze data is the high heterogeneity of criteria used for assessing intracranial response in BMs. Slightly more than 25% of the studies reported using the RANO-BM criteria [99], which should represent the gold standard. The remainder predominantly saw the use of RECIST 1.1 or RECIST 1.0 criteria or the investigator’s choice. It would be ideal to report stratified data for patients with stable and active BMs, as this would improve result clarity and refine clinical indications. This stratification is essential for distinguishing the causal impact of intracranial responses due to systemic therapy, prior local therapy, or the interplay between both treatment modalities. Additionally, a key factor in interpreting intracranial response data is the standardization of eligibility criteria, particularly regarding the washout period from the last local therapy in cases of pretreated BMs.

The data gathered in this review demonstrate remarkable consistency for HER2- + BCBM and provide valuable guidance for clinical decision-making. Whether pretreated or not, the outstanding efficacy of T-DXd in stable HER-2 + BMs suggests it may be preferred over other active regimens such as T-DM1 or the Trastuzumab–Tucatinib–Capecitabine combination [74,78,116]. However, despite encouraging findings from the Destiny breast-12, DEBBRAH, and TUXEDO trials, the evidence remains too premature to recommend T-DXd for patients with active or progressing brain metastases. Ideally, a randomized trial would be the gold standard with which to determine whether systemic therapy with ADCs alone in BMs can achieve superior outcomes, potentially delaying or even reducing the need for local interventions.

## 6. Conclusions

ADCs are ready to become a key component of clinical practice for BC and NSCLC with active or stable brain metastases, offering the potential to delay other systemic or local therapies while improving survival outcomes. In pBTs, the landscape for ADCs remains more complex due to the unique biological challenges of these diseases. However, advancements in next-generation ADCs are likely to overcome these obstacles, paving the way for more effective treatment strategies in both primary and metastatic brain tumors.

## Figures and Tables

**Table 1:** FDA-approved ADCs for solid tumors.

ADC	Target	Linker Type	Payload	Indications	Registration Trial
Mirvetuximab soravtansine	Folate receptor alpha	Cleavable	Maytansinoid DM4	Folate receptor-alpha (FRα)-positive, platinum-resistant high-grade serous epithelial ovarian, fallopian tube, or primary peritoneal cancer patients who have received 1 to 3 prior systemic treatment regimens	SORAYA (NCT04296890)
Tisotumab vedotin	Tissue Factor	Cleavable	Monomethyl Auristatin E (MMAE)	Recurrent or metastatic cervical cancer with disease progression to chemotherapy	InnovaTV 301 (NCT04697628)
Ado-trastuzumab emtansine	HER2	Non cleavable	Maytansine DM1	EBC: Adjuvant treatment of patients with HER2-positive EBC who have residual invasive disease after neoadjuvant taxane- and trastuzumab-based treatment. MBC: Patients with HER2-positive MBC who previously received trastuzumab and a taxane, separately or in combination	KATHERINE (NCT01772472) EMILIA (NCT00829166)
Enfortumab vedotin	Nectin-4	Clevable	Monomethyl Auristatin E (MMAE)	Adult patients with locally advanced or metastatic urothelial cancer who have received an anti PD-(L)1 agent, and platinum-based chemotherapy	EV-301 (NCT03474107)
Trastuzumab deruxtecan	HER2	Cleavable	Deruxtecan	**Breast cancer:** Unresectable or metastatic HER2-positive (IHC 3+ or ISH positive) BC patients who have received a prior anti-HER2-based regimen Patients with unresectable or metastatic HER2-low (IHC 1+ or IHC 2+/ISH−) BC, as determined by an FDA-approved test, who have received a prior chemotherapy in the metastatic setting **Non-small cell lung cancer:** Unresectable or metastatic NSCLC patients whose tumors have activating HER2 mutations, as detected by an FDA-approved test, and who have received a prior systemic therapy (accelerated approval) **Gastric cancer:** Locally advanced or metastatic HER2-positive (IHC 3+ or IHC 2+/ISH positive) gastric or gastroesophageal junction (GEJ) adenocarcinoma patients who have received a prior trastuzumab-based regimen **Solid tumors:** Patients with unresectable or metastatic HER2-positive (IHC3+) solid tumors who have received prior systemic treatment (accelerated approval)	DESTINY-Breast03 (NCT03529110) DESTINY-Breast04 (NCT03734029) DESTINY-Lung02 (NCT04644237) DESTINY-Gastric01 (NCT03329690) DESTINY-PanTumor02 (NCT04482309)
Sacituzumab Govitecan	TROP2	Cleavable	Camptothecin analog (SN38)	**BC:** Unresectable locally advanced or metastatic triple-negative BC patients who have received 2 or more prior systemic therapies **Urothelial cancer:** Locally advanced or metastatic urothelial cancer (mUC) patients who have previously received platinum-based chemotherapy and an anti PD-(L)1 inhibitor. (accelerated approval)	ASCENT (NCT02574455) IMMU-132-01 (NCT01631552) TROPHY (NCT03547973)
Datopotomab Deruxtecan	TROP2	Cleavable	Topoisomerase I inhibitor (Deruxtecan)	Patients with unresectable or metastatic hormone receptor-positive, HER2-breast cancer that has progressed after endocrine-based therapy and chemotherapy for unresectable or metastatic disease	TROPION-Breast01 (NCT05104866)

Abbreviations: ADC, antibody–drug conjugates; BC, breast cancer; DM1, Maytansinoid Derivative 1; EBC, Early breast cancer; DM4, Maytansinoid Derivative 4; EBC, early breast cancer; FRα, folate receptor-alpha; HER2, human epidermal growth factor receptor 2; IHC, immunohistochemistry; ISH, in situ hybridization; MBC, metastatic breast cancer; MMAE, monomethyl auristatin E; NCT, National Clinical Trial; NSCLC, non-small cell lung cancer; PD-1, Programmed Death-1; PD-L1, Programmed Death-Ligand 1; SN38, Camptothecin analog, the active metabolite of irinotecan.

**Table 3:** Summary of ongoing clinical trial evaluating ADC in primary brain tumors patients.

Clinical Trial	Response Endpoint	Treatment	Target	Linker Type	Payload Action	Population	Trial Type	Status
AMG 595 First-in-Human in Recurrent Gliomas (NCT01475006)	ORR by Macdonald criteria	AMG 595	EGFRvIII	Non-Cleavable	Microtubule inhibitor (Maytansinoid DM1)	Patients with rGBM (Part 1 and 2) or WHO Grade III anaplastic astrocytoma (Part 1 only) expressing EGFRvIII	Phase I	Completed. No results published (last update 2016)
Safety and Efficacy of L19TNF in Patients with Isocitrate Dehydrogenase (IDH) Wildtype WHO Grade III/IV Glioma at First Relapse (GLIOMOON) (NCT03779230)	PFS, OS, ORR by iRANO criteria	Onfekafusp alfa (L19TNF)	Fibronectin	NA	TNFalpha	Patients with WHO Grade III/IV Glioma at First Relapse	Phase I/II	Completed
Safety and Efficacy of L19TNF Plus Temozolomide Chemoradiotherapy in Patients with Newly Diagnosed Glioblastoma (GLIOSUN) (NCT04443010)	OS, PFS, ORR, DCR, BORR by iRANO	Onfekafusp alfa (L19TNF) + TMZ	Fibronectin	NA	TNFalpha	Patients with newly diagnosed GBM	Phase II	Recruiting
A Study to Evaluate Safety and Efficacy of L19TNF Plus Lomustine in Patients with Glioblastoma at First Progression (GLIOSTAR) (NCT04573192)	OS, PFS by iRANO	Onfekafusp alfa (L19TNF) + lomustine	Fibronectin	NA	TNFalpha	Patients with GBM at first progression	Phase I/II	Recruiting
Neuro/Sacituzumab Govitecan/Breast Brain Metastasis/Glioblastoma/Ph 0 (NCT03995706)	Ratio of SN-38 and its metabolites relative to serum concentration	Sacituzumab Govitecan	TROP2	Cleavable	Topoisomerase I Inhibitor (govitecan)	Patients with rGBM/BM from breast	Phase 0	Completed

Abbreviations: ADC, antibody–drug conjugate; BM, brain metastasis; BORR, best overall response rate; DCR, disease control rate; EGFRvIII, epidermal growth factor receptor variant III; iRANO, Immunotherapy Response Assessment in Neuro-Oncology; ORR, overall response rate; OS, overall survival; PFS, progression-free survival; TNFalpha, tumor necrosis factor alfa; TROP2, trophoblast cell surface antigen 2.

**Table 4:** Summary of available clinical trials of ADCs in NSCLC with data on intracranial activity.

Clinical Trial	Type of Trial	ADC	Target	Linker Type	Payload Action	Population	CNS Metastasis Eligibility	Intracranial Activity	Intracranic Response Criteria
TROPION-Lung05 (NCT04484142)	Phase II	Datopotamab Deruxtecan (Dato-DXd)	TROP-2	Cleavable	Topoisomerase I inhibitor (Deruxtecan)	a/m NSCLC with AGAs who progressed following targeted therapy and platinum-based CT	Clinically inactive BM (not requiring medical therapy) eligible. Leptomeningeal disease excluded	iORR 22% iDCR 72%	RECIST v1.1
DESTINY-Lung01 (NCT03505710) DESTINY-Lung02 (NCT04644237)	Phase II	Trastuzumab-Deruxtecan (T-DXd)	HER2	Cleavable	Topoisomerase I inhibitor (Deruxtecan)	HER2-overexpressing or HER2-mutant unresectable or metastatic NSCLC	No exclusion criteria for patients with brain metastases, including leptomeningeal carcinomatosis	5.4 mg/kg Cohort (DL-02): iORR 25% iDCR 81.3% 6.4 mg/kg Cohort (DL-01/02): iORR 18.5% iDCR 76%	RECIST v1.1
HERTHENA-Lung01 (NCT04619004)	Phase II	Patritumab Deruxtecan (HER3-DXd)	HER3	Cleavable	Topoisomerase I inhibitor (Deruxtecan)	a/m NSCLC with canonical EGFR mutation who progressed on at least one EGFR TKI and one platinum-based CT	Clinically inactive BM (not requiring medical therapy) eligible. Leptomeningeal disease excluded	iORR 33.3% iDCR 76.6%	RECIST v1.1

Abbreviations: a/m, advanced/metastatic; ADC, antibody–drug conjugate; AGAs, actionable genomic alterations; CT, chemotherapy; Dxd, Deruxtecan; HER2, human epidermal growth factor receptor 2; HER3, human epidermal growth factor receptor 3; iDCR, intracranial disease control rate; iORR, intracranial overall response rate; NSCLC, non-small cell lung cancer; TROP-2, trophoblast cell-surface antigen 2.

**Table 5:** Summary of available clinical trials of ADCs in BC with data on intracranial activity.

Clinical Trial	Type of Trial	ADC	Target	Linker Type	Payload Action	Population	CNS Metastasis Eligibility	CNS Endpoints	Intracranic Response Criteria
EMILIA (NCT008291)	Retrospective exploratory analysis	Trastuzumab emtansine (T-DM1)	HER2	Non-cleavable	Microtubule Inhibitor (Maytansinoid DM1)	HER2-positive a/m BC, previously treated with trastuzumab and a taxane	Treated, asymptomatic	Patients with baseline BM mOS: 26.8 vs. 12.9 mo	RECIST v1.1
TH3RESA (NCT014191)	Phase III	Trastuzumab emtansine (T-DM1)	HER2	Non-cleavable	Microtubule Inhibitor (Maytansinoid DM1)	HER2-positive mBC pretreated with ≥2 HER2 directed regimens	Treated, asymptomatic	Patients with baseline BM mPFS: 5.8 vs. 2.9 mo	RECIST v1.1
KAMILLA (NCT017025)	Exploratory analysis	Trastuzumab emtansine (T-DM1)	HER2	Non-cleavable	Microtubule Inhibitor (Maytansinoid DM1)	HER2-positive a/m BC progressing after CT and anti-HER2 therapy or ≤6 mo after adjuvant therapy	Controlled or untreated and asymptomatic	iORR: 21.4%, iCR: 2.4%	RECIST v1.1
DESTINY-Breast01 (NCT032484)	Phase II	Trastuzumab Deruxtecan (T-DXd)	HER2	Cleavable	Topoisomerase I Inhibitor (Deruxtecan)	HER2-positive mBC previously treated with T-DM1	Treated, asymptomatic	iORR: 41.2%	RECIST v1.1
DESTINY-Breast03 (NCT035291)	Phase III	Trastuzumab Deruxtecan (T-DXd)	HER2	Cleavable	Topoisomerase I Inhibitor (Deruxtecan)	HER2-positive mBC previously treated with trastuzumab and a taxane	Treated or asymptomatic	iORR: 79 vs. 35%	RECIST v1.1
TUXEDO-1 (NCT047520)	Phase II	Trastuzumab Deruxtecan (T-DXd)	HER2	Cleavable	Topoisomerase I Inhibitor (Deruxtecan)	HER2-positive mBC and newly diagnosed untreated BM/BM progressing after previous local therapy, previous exposure to trastuzumab and pertuzumab and no indication for immediate local therapy	Newly diagnosed untreated/progressing after previous local therapy	iORR: 73.3% iCR: 13.3% iPR: 60% iSD: 20%	RANO-BM
DESTINY-Breast12 (NCT047397)	Phase IIIb/IV	Trastuzumab- Deruxtecan (T-DXd)	HER2	Cleavable	Topoisomerase I Inhibitor (Deruxtecan)	HER2-positive mBC treated with one or more prior anti-HER2–based regimens	Stable or active (untreated/previously treated and progressing)	iORR: 71.7% (79.2% with stable BM and 62.3% with active BM)	RECIST v1.1
DEBBRAH (NCT044205)	Phase II	Trastuzumab- Deruxtecan (T-DXd)	HER2	Cleavable	Topoisomerase I Inhibitor (Deruxtecan)	HER2-positive or HER-2 low a/m BC pretreated	Cohort 2: asymptomatic, untreated Cohort 3: progressing after local therapy	CNS ORR: 50% (cohort 2) 44.4% (cohort 3)	RANO-BM
ASCENT (NCT025744)	Subgroup analysis	Sacituzumab Govitecan (SG)	TROP2	Cleavable	Topoisomerase I Inhibitor (govitecan)	relapsed or refractory mTNBC	Stable	mOS in BM population: 6.8 vs. 7.5 mo	RECIST v1.1

Abbreviations: a/m, advanced/metastatic; ADC, antibody–drug conjugate; BC, breast cancer; BM, brain metastases; CT, chemotherapy; Dxd, Deruxtecan; HER2, human epidermal growth factor receptor 2; iORR, intracranial overall response rate; iCR, intracranial complete response; iPR, intracranial partial response; iSD, intracranial stable disease; mOS, median overall survival; mPFS, median progression-free survival; mTNBC, metastatic triple-negative breast cancer; mo, months; TROP2, trophoblast cell-surface antigen 2.

**Table 6:** Summary of ongoing clinical trials evaluating ADC in mBC patients with BM.

Clinical Trial	Response Endpoint	ADC	Target	Linker Type	Payload Action	Population	CNS Metastasis Eligibility	Trial Type	Status
DATO-BASE: DATOpotamab-deruxtecan for Breast Cancer Brain MetAstaSEs (NCT06176261)	iORR as defined by RANO-BM criteria	Datopotamab Deruxtecan (Dato-DXd)	TROP2	Cleavable	Topoisomerase I Inhibitor (Deruxtecan)	HER2-negative mBC	Cohorts A and B: newly diagnosed or progressing after local and/or systemic therapy Cohort C: leptomeningeal disease	Phase II	Recruiting
T-DXd Therapy for HER2-low Breast Cancer Patients with Brain Metastases (TUXEDO-4) (NCT06048718)	iORR as defined by RANO-BM criteria	Trastuzumab Deruxtecan (T-DXd)	HER2	Cleavable	Topoisomerase I Inhibitor (Deruxtecan)	HER2-low mBC with newly diagnosed or progressing Bm with or without untreated type II LMD, who received ≥1 line of systemic treatment in the advanced setting	Newly diagnosed or progressive, without indication for immediate local therapy	Phase II	Recruiting
Testing Sacituzumab Govitecan Therapy in Patients With HER2-Negative Breast Cancer and Brain Metastases (NCT04647916)	iORR as defined by RANO-BM criteria	Sacituzumab govitecan (SG)	TROP2	Cleavable	Topoisomerase I Inhibitor (govitecan)	HER2-negative mBC with BM	≥1 BM not previously treated or progressed to RT	Phase II	Recruiting
HER3-DXd in Breast Cancer and NSCLC Brain Metastases and Solid Tumor Leptomeningeal Disease (TUXEDO-3) (NCT05865990)	iORR as defined by RANO-BM criteria	Patritumab deruxtecan (HER3-DXd)	HER3	Cleavable	Topoisomerase I Inhibitor (Deruxtecan)	mBC or aNSCLC with active BM), who received ≥1 line of systemic treatment in the advanced setting or with active LMD after RT from an advanced solid tumor who do not need immediate local treatment, and have not received prior treatment with an anti-HER3	Active BM from MBC (Cohort 1) and aNSCLC (Cohort 2), untreated LMD (Cohort 3)	Phase II	Active, not recruiting
Patritumab Deruxtecan (U3-1402) in Unresectable Locally Advanced or Metastatic Breast Cancer (ICARUS BREAST) (NCT04965766)	ORRas defined by RECIST	Patritumab deruxtecan (HER3-DXd)	HER3	Clevable	Topoisomerase I Inhibitor (Deruxtecan)	HR+, ABC, resistant to ET and CDK4/6i, that must have received only one line of chemotherapy for ABC	Clinically inactive, treated, asymptomatic	Phase II	Active, not recruiting
A Phase 1b/2 Study of T-DXd Combinations in HER2-positive Metastatic Breast Cancer (DESTINY-Breast07) (NCT04538742)	ORR, PFS, PFS2, DoR as defined by RECIST, OS	Trastuzumab Deruxtecan (T-DXd)	HER2	Cleavable	Topoisomerase I Inhibitor (Deruxtecan)	HER2-positive a/mBC, ≥1 line of systemic treatment in the advanced setting (Part 1), no prior lines for a/mBC (Part 2 Modules 0–5), zero or one prior lines for a/mBC allowed (Part 2 Modules 6 and 7	Stable (Modules 0–5); untreated, not needing local therapy or previously treated that have progressed since prior local therapy (Module 6 and 7) (<2 mg dexamethasone/day)	Phase Ib/II	Active, not recruiting
PRE-I-SPY Phase I/Ib Oncology Platform Program (PRE-I-SPY-PI) (NCT05868226)	ORR, DoR, PFS, CBR as defined by RECIST	Trastuzumab Deruxtecan (T-DXd)	HER2	Cleavable	Topoisomerase I Inhibitor (Deruxtecan)	HER2 expressing (>HER2 1+) mBC	Treated and clinically stable	Phase I/Ib	Recruiting

Abbreviations: ABC, advanced breast cancer; ADC, antibody–drug conjugate; a/m, advanced/metastatic; aNSCLC, advanced non-small cell lung cancer; BM, brain metastasis; CBR, clinical benefit rate; CDK4/6i, cyclin-dependent kinases 4 and 6 inhibitors; DoR, duration of response; Dxd, Deruxtecan; ET, endocrine therapy; HER2, human epidermal growth factor receptor 2; HER3, human epidermal growth factor receptor 3; HR+, hormone receptor-positive; iORR, intracranial overall response rate; LMD, leptomeningeal disease; OS, overall survival; PFS, progression-free survival; RT, radiotherapy; TROP2, trophoblast cell-surface antigen 2.

## Appendix A

**Table A1:** Summary of ongoing clinical trials evaluating ADCs in NSCLC patients.

Clinical Trial	Response Endpoint	ADC	Target	Linker Type	Payload Action	Population	CNS Metastasis Eligibility	Trial Type	Status
HER3-DXd in Breast Cancer and NSCLC Brain Metastases and Solid Tumor Leptomeningeal Disease (TUXEDO-3) (NCT05865990)	iORR as defined by RANO -BM criteria	Patritumab deruxtecan (HER3-DXd)	HER3	Cleavable	Topoisomerase I inhibitor (Deruxtecan)	Patients with previously treated a/m NSCLC with newly diagnosed or progressive BM	Patients are eligible if steroid use is <8 mg of Dexamethasona/day	Phase II	Active, not recruiting
Datopotamab Deruxtecan (Dato-DXd) for Non-Small Cell Lung Cancer (NSCLC) patients with Active Brain Metastases (TUXEDO-5) (NCT06676917)	iORR as defined by RANO-BM criteria	Datopotamab Deruxtecan (Dato-DXd)	TROP2	Cleavable	Topoisomerase I inhibitor (Deruxtecan)	Patients with previously treated a/m non squamous NSCLC with active BM	Patients with BM and leptomeningeal disease are allowed if there is no indication for immediate local therapy	Phase II	Recruitment not yet open; the estimated study start date is April 2025
Sacituzumab Tirumotecan (MK-2870) Versus Chemotherapy in Previously Treated a/m Nonsquamous NSCLC with EGFR Mutations or Other Genomic Alterations (NCT06074588)	PFS as defined by RECIST v 1.1 and OS	Sacituzumab tirumotecan (MK-2870)	TROP2	Cleavable	Topoisomerase I Inhibitor (Tirumotecan)	Patients with a/m non-squamous NSCLC with treatment-refractory progressive disease	Previously treated BM included; active BM and leptomeningeal disease excluded	Phase III	Recruiting
Phase III, Open-label, First-line Study of Dato-DXd in Combination with Durvalumab and Carboplatin for Advanced NSCLC Without Actionable Genomic Alterations (AVANZAR) (NCT05687266)	PFS as defined by RECIST v1.1 and OS	Datopotamab Deruxtecan (Dato-DXd)	TROP2	Clevable	Topoisomerase I inhibitor (Deruxtecan)	Patients with a/m NSCLC without actionable genomic alterations with approved and a viable target therapy	Active BM and leptomeningeal disease excluded	Phase III	Active, not recruiting
Study of Pembrolizumab (MK-3475) Monotherapy Versus Sacituzumab Govitecan in Combination with Pembrolizumab for Participants With Metastatic Non-small Cell Lung Cancer (NSCLC) With Programmed Cell Death Ligand 1 (PD-L1) Tumor Proportion Score (TPS) ≥ 50% (MK-3475-D46) (NCT05609968)	PFS as defined by RECIST v1.1 and OS	Sacituzumab govitecan (SG)	TROP2	Cleavable	Topoisomerase I Inhibitor (govitecan)	Patients with a/m NSCLC without an indication of EGFR-ALK-1, or ROS-1-targeted therapies with PD-L1 expression > 50%	Active BM and leptomeningeal disease excluded	Phase III	Recruiting
Study of Dato-DXd Plus Pembrolizumab vs. Pembrolizumab Alone in the First-line Treatment of Subjects with Advanced or Metastatic NSCLC Without Actionable Genomic Alterations (TROPION-Lung08) (NCT05215340)	PFS as defined by RECIST v1.1 and OS	Datopotamab Deruxtecan (Dato-DXd)	TROP2	Cleavable	Topoisomerase I Inhibitor (Deruxtecan)	Patients with a/m non-squamous NSCLC without actionable genomic alterations (mKRAS eligible) with PD-L1 expression > 50%	Active and untreated BM excluded; leptomeningeal disease excluded	Phase III	Recruiting
A Study of YL202 in Selected Patients with Advanced Solid Tumors (NCT06107686)	ORR as defined by RECIST v1.1	YL202	HER3	Cleavable	Topoisomerase I Inhibitor (YL0010014)	Patients with a/m NSCLC	Patients with BM excluded	Phase II	Recruiting
A Study of Disitamab Vedotin in Previously Treated Solid Tumors That Express HER2 (NCT06003231)	ORR as defined by RECIST v1.1	Disitamab Vedotin	HER2	Cleavable	Auristatin microtubule inhibitor (monomethyl auristatin E)	Patients with a/m solid tumors, including NSCLC, with HER2 overexpression	Active and untreated BM and leptomeningeal disease excluded	Phase II	Recruiting
A Phase 2 Study of BA3011 Alone and in Combination with PD-1 Inhibitor in Adult Patients with Metastatic Non-small Cell Lung Cancer (NSCLC) Who Had Prior Disease Progression on a PD-1/L-1 Inhibitor, EGFR, or ALK Inhibitor. (NCT04681131)	ORR as defined by RECIST v1.1	CAB-AXL-ADC	AXL	Cleavable	Auristatin microtubule inhibitor (monomethyl auristatin E)	Patients with a/m NSCLC	Patients with active BM excluded	Phase II	Active, non recruiting
Datopotamab Deruxtecan (Dato-DXd, DS-1062a) in Advanced and/or Unresectable Non-Small Cell Lung Cancer (ICARUS-LUNG01) (NCT04940325)	ORR as defined by RECIST v1.1	Datopotomab Deruxtecan (Dato-DXd)	TROP2	Cleavable	Topoisomerase I Inhibitor (Deruxtecan)	Patients with a/m NSCLC	BM eligible only if treated and clinically stable without medical treatment	Phase II	Active, non recruiting
CAB-ROR2-ADC Safety and Efficacy Study in Patients with TNBC or Head & Neck Cancer (Ph1) and NSCLC or Melanoma (Ph2) (NCT03504488)	ORR	Ozuriftamab vedotin	ROR2	Cleavable	Auristatin microtubule inhibitor (monomethyl auristatin E)	Patients with a/m solid tumors, including NSCLC, that is refractory or not amenable to the standard-of-care therapy	Uncontrolled BM excluded	Phase I/II	Active, non recruiting
A Clinical Trial of TQB2102 for Injection in Non-small Cell Lung Cancer with HER2 Gene Abnormality (NCT06496490)	ORR	TQB2102	HER2	Cleavable	Topoisomerase I Inhibitor (topotecan)	Patients with a/m NSCLC refractory to standard-of-care therapy	Stable BM included Uncontrolled BM or Leptomeningeal disease excluded	Phase II	Recruiting
Study to Investigate Luveltamab Tazevibulin in Adults with Advanced or Metastatic Non-small Cell Lung Cancer (NCT06555263)	ORR as defined by RECIST v1.1	Luveltamab Tazevibulin	FOLR1	Cleavable	Auristatin microtubule inhibitor (hemiasterina)	Patients who are a/m NSCLC-positive per FOLR1 expression per central testing	Untreated BM are excluded	Phase II	Recruiting
A First-in-human Study Using BDC-1001 as a Single Agent and in Combination with Nivolumab in Advanced HER2-Expressing Solid Tumors (NCT04278144)	ORR	BDC-1001	HER2	Non cleavable	ISAC (Trastuzumab conjugated with TLR7/8 agonist)	Patients with an a/m solid tumor with documented HER2-protein expression or gene amplification for which approved therapies have been exhausted or not clinically indicated	Untreated BM or active BM are excluded	Phase I/II	Active, non recruiting
Phase I–II, FIH, TROP2 ADC, Advanced Unresectable/Metastatic Solid Tumors, Refractory to Standard Therapies (A264) (NCT04152499)	ORR (phase II)	SKB264	TROP2	Cleavable	Microtubule inhibitor (Belotecan-based payload)	Patients with a/m solid tumors, including NSCLC, that is refractory or not amenable to the standard-of care-therapy	Symptomatic BM or any radiation or surgery for BMs within 1 month of first infusion of study drug are the exclusion criteria (Phase I). Patients with active BM or leptomeningeal disease are excluded (Phase II)	Phase I/II	Recruiting
PRO1184 for Advanced Solid Tumors (PRO1184-001) (NCT05579366)	ORR as defined by Recist v1.1 (phase II)	PRO1184	FOLR1	Cleavable	Topoisomerase I Inhibitor (exatecan)	Patients with a/m solid tumors, including NSCLC, that is refractory or not amenable to the standard-of-care therapy	Active BMs are excluded	Phase I/II	Recruiting
Study of DF1001 in Patients with Advanced Solid Tumors (NCT04143711)	ORR as defined by RECIST v1.1	DF1001	HER2	Cleavable	Induce cell death by targeting NK cells and T-cell activation signals	A/m NSCLC patients with HER2 overexpression/amplification/mutation who progressed after a platinum-based CT and have received and progressed on or after anti PD-(L)1 therapy. Those for whom a/m NSCLC progressed during or after platinum doublet-based chemotherapy (expansion NSCLC cohort)	Active BMs or a history of BM are the exclusion criteria	Phase I/II	Recruiting
Study of REGN5093-M114 (METxMET Antibody–Drug Conjugate) in Adult Patients with Mesenchymal–Epithelial Transition Factor (MET) Overexpressing Advanced Cancer (NCT04982224)	ORR (Phase II)	REGN5093-M114	METxMET	Cleavable	Auristatin microtubule inhibitor (M24)	Patients with a/m NSCLC with MET Overexpression	Untreated/active BM and leptomeningeal disease excluded	Phase I/II	Recruiting
Clinical Study of Antibody–Drug Conjugate MYTX-011 in Subjects with Non-Small Cell Lung Cancer (NCT05652868)	ORR, OS and PFS are secondary endpoints	MYTX-011	MET	Clevable	Auristatin microtubule inhibitor (Monomethyl auristatin E)	Patients with a/m NSCLC with MET alterations that is refractory to the standard-of-care therapy	Untreated/active BM and leptomeningeal disease excluded	Phase I	Recruiting
An Efficacy and Safety Study of Cofetuzumab Pelidotin in Participants with PTK7-Expressing, Recurrent Non-Small Cell Lung Cancer (NCT04189614)	OS, PFS and DOR are secondary endpoints	Cofetuzumab pelidotin	PTK7	Cleavable	Auristatin microtubule inhibitor (Auristatin-0101)	Patients with recurrent and treatment-refractory NSCLC with PTK7 expression	Asymptomatic and treated BM included, active BM excluded	Phase I	Active, non recruiting
A Study to Evaluate TROP2 ADC LCB84 Single Agent and in Combination with an Anti-PD-1 Ab in Advanced Solid Tumors (NCT05941507)	PFS, ORR, DOR, TTP as defined by RECIST v1.1, iRECIST, and RANO-BM OS	LCB84	TROP2	Cleavable	Microtubule inhibitor (Monomethyl auristatin E)	Patients with advanced solid tumors, including NSCLC that is refractory or not amenable to the standard-of-care therapy	Stable and treated BM included	Phase I/II	Recruiting
DS8201a and Pembrolizumab in Participants with Locally Advanced/Metastatic Breast or Non-Small Cell Lung Cancer (NCT04042701)	ORR	Trastuzumab deruxtecan	HER2	Cleavable	Topoisomerase I Inhibitor (Deruxtecan)	Patients with a/m breast cancer or NSCLC with HER2 overexpression or HER2-mutant disease	Active BM excluded	Phase I	Recruiting
Phase Ib Study of the Safety of T-DXd and Immunotherapy Agents with and Without Chemotherapy in Advanced or Metastatic HER2-positive, non-squamous NSCLC (DL03) (NCT04686305)	PFS, ORR, DOR, as defined by RECIST v1.1, OS	Trastuzumab Deruxtecan (T-DXd)	HER2	Cleavable	Topoisomerase I Inhibitor (Deruxtecan)	Patients with a/m non-squamous NSCLC that are refractory to systemic therapy or treatment-naive with HER2 overexpression	Untreated and symptomatic BM excluded	Phase I	Recruiting
A Study of LY4052031 in Participants with Advanced or Metastatic Urothelial Cancer or Other Solid Tumors (NEXUS-01) (NCT06465069)	ORR defined by RECIST v1.1	LY4052031	NECTIN4	Cleavable	Topoisomerase I Inhibitor (Camptothecin-98)	Patients with a/m solid tumors, including NSCLC that is refractory or not amenable to the standard-of-care therapy	Known or suspected uncontrolled BM excluded	Phase I	Recruiting
A Study of LY4170156 in Participants with Selected Advanced Solid Tumors (NCT06400472)	ORR defined by RECIST v1.1	LY4170156	FOLR1	Cleavable	Topoisomerase I Inhibitor (Camptothecin-98)	Patients with advanced solid tumors, including NSCLC that is refractory or not amenable to the standard-of-care therapy	Active and untreated BM, and history of leptomeningeal disease excluded	Phase I	Recruiting
A Study of LY4101174 in Participants with Recurrent, Advanced or Metastatic Solid Tumors (NCT06238479)	ORR defined by RECIST v1.1 (primary endpoint) DOR, PFS, TTR, DCF as defined by RECIST v1.1 OS	LY4101174	NECTIN4	Cleavable	Topoisomerase I Inhibitor (Camptothecin-98)	Patients with a/m solid tumors, including NSCLC that is refractory or not amenable to the standard-of-care therapy	Known or suspected uncontrolled BM excluded	Phase I	Recruiting
Safety of GQ1001 in Adult Patients with HER2-Positive Advanced Solid Tumors (NCT04450732)	ORR, DCR, PFS and DoR as defined by RECIST v1.1	GQ1001	HER2	Cleavable	Microtubule inhibitor (Maytansinoide DM1)	Patients with a/m HER2-expressing solid tumors, including NSCLC that is refractory or not amenable to the standard-of-care therapy	Treated and asymptomatic BM included untreated and symptomatic BM excluded	Phase I	Recruiting
Phase I–II, FIH, TROP2 ADC, Advanced Unresectable/Metastatic Solid Tumors, Refractory to Standard Therapies (A264) (NCT04152499)	ORR, DoR, PFS as defined by RECIST v1.1 OS	Sacituzumab tirumotecan	TROP2	Cleavable	Topoisomerase I inhibitor (belotecan derivative)	Patients with a/m solid tumors, including metastatic NSCLC, that is refractory or not amenable to the standard of-care-therapy	Symptomatic and active BM, history of LMD, brainstem metastasis, and spinal cord metastasis excluded	Phase I/II	Recruiting
Phase 1 Study of CPO301 in Adult Patients with Advanced or Metastatic Solid Tumors (NCT05948865)	Efficacy assesment not specified	CPO301	EGFR	Cleavable	Non-specified	Patients with a/m solid tumors, including metastatic NSCLC, that is refractory or not amenable to the standard-of-care therapy	Known, active, or uncontrolled BM or leptomeningeal disease excluded	Phase I	Recruiting
A Study of PF-08046050 (SGN-CEACAM5C) in Adults with Advanced Solid Tumors (NCT06131840)	ORR, DoR, PFS as defined by RECIST v1.1 Best response OS	PF-08046050	CEACAM5	Cleavable	Topoisomerase I inhibitor	Patients with a/m solid tumors, including metastatic NSCLC, that is refractory or not amenable to the standard-of-care therapy	Stable and treated BM included	Phase I	Recruiting
PRO1184 for Advanced Solid Tumors (PRO1184-001) (NCT05579366)	ORR, DoR, PFS as defined by RECIST v1.1 OS	PRO1184	FOLR1	Cleavable	Topoisomerase I inhibitor	Patients with a/m solid tumors, including NSCLC that is refractory or not amenable to the standard-of-care therapy	Previously treated and stable BM included active BM or leptomeningeal diseaseexcluded	Phase I/II	Recruiting

Abbreviations: ADC, antibody–drug conjugate; a/m, advanced/metastatic; AXL, AXL receptor tyrosine kinase; BM, brain metastasis; CEACAM 5, Carcinoembryonic Antigen-Related Cell Adhesion Molecule 5; DCR, disease control rate; DoR, duration of response; Dxd, Deruxtecan; EGFR, epidermal growth factor receptor; FOLR1, Folate Receptor Alpha; HER2, human epidermal growth factor receptor 2; HER3, human epidermal growth factor receptor 3; iDCR, intracranial disease control rate; iORR, intracranial overall response rate; MET, mesenchymal–epithelial transition factor; Nectin 4, Nectin Cell Adhesion Molecule 4; NSCLC, non-small cell lung cancer; OS, overall survival; PFS, progression-free survival; PTK7, Protein Tyrosine Kinase 7; ROR2, receptor tyrosine kinase-like orphan receptor 2; TF, tissue factor; TROP2, trophoblast cell-surface antigen 2.
